# Regulatory Role of Zinc in Acute Promyelocytic Leukemia: Cellular and Molecular Aspects with Therapeutic Implications

**DOI:** 10.3390/ijms26199685

**Published:** 2025-10-04

**Authors:** Norihiro Ikegami, István Szegedi, Csongor Kiss, Miklós Petrás

**Affiliations:** Division of Pediatric Hematology-Oncology, Department of Pediatrics, Faculty of Medicine, University of Debrecen, 4032 Debrecen, Hungary

**Keywords:** zinc, acute promyelocytic leukemia, arsenic trioxide, all-trans retinoic acid

## Abstract

Acute promyelocytic leukemia (APL) is a rare subtype of acute myeloid leukemia (AML) characterized by chromosomal translocation forming the fusion protein that blocks the differentiation of myeloid progenitors and increases the self-renewal of leukemia cells. The introduction of all-trans retinoic acid (ATRA) and arsenic trioxide (ATO) has dramatically improved outcomes in APL, making it a leading example of successful treatment through differentiation of cancer cells. However, life-threatening side effects and treatment resistance may develop; therefore, modulation of the safety and efficacy of these drugs may contribute to further improving treatment results. Recently, zinc, involved in the structure and function of transcription factors, has received special attention for its potential role in the development and treatment response of cancer. Zinc homeostasis is disrupted in APL, with intracellular accumulation stabilizing oncogenic proteins. Zinc depletion promotes degradation of PML–RARA and induces apoptosis, while supplementation enhances genotoxic stress in leukemic cells but protects normal hematopoiesis. Zinc also regulates key transcription factors involved in differentiation and proliferation, including RUNX2, KLF4, GFI1, and CREB. In this review, we examine how zinc may impact zinc-finger (ZnF) and non-ZnF transcription factors and differentiation therapy in APL, thereby identifying potential strategies to enhance treatment efficacy and minimize side effects.

## 1. Introduction

Acute myeloid leukemia (AML) is a hematopoietic stem cell disorder characterized by the clonal proliferation of abnormally differentiated myeloid lineage blasts [1]. Globally, the proportion of AML among all leukemia cases was observed to increase significantly from 18.0% to 23.1% between 1990 and 2017, while the age-standardized incidence rate (ASIR) also increased from 1.35/100,000 to 1.54/100,000 in this period [2]. Acute promyelocytic leukemia (APL) is a rare and uniform subtype of AML, representing about 5–20% of all AML cases. The annual incidence is estimated to be between 1 to 7.4 cases per 1,000,000. While it is a rare disease in children overall, its incidence can vary geographically, with some regions, like Latin America, Southern Europe and Africa, reporting higher proportions of APL among childhood AML. APL incidence peaks in infants under one year of age and then declines, with a slight increase again in adolescents [3]. Within the national population of 10 million in Hungary, there are 10 to 12 new pediatric AML cases annually. From 2012 to 2019, a total of 92 children and adolescents aged 0.1 to 19 years were diagnosed with newly developed AML. Of these, 10 cases were APL [4].

The cure of AML remains challenging due to its significant heterogeneity, high risk of relapse, and the toxicity of treatment [1]. Since the 1980s, overall survival (OS) rates for pediatric AML have improved dramatically, reaching up to 75% in developed countries. However, this rate still falls short of the approximately 90% OS achieved in pediatric acute lymphoblastic leukemia (ALL), thanks to major advances in personalized and targeted therapies driven by a deeper understanding of its molecular mechanisms. Furthermore, nearly half of pediatric AML patients experience relapse, and their outcomes remain poor. Notably, survival rates of children with AML vary significantly even within some regions in Europe, where they are below 50%, as reflected by differences in access to specialized care and resources [1,5]. In Hungary, the 4-year OS rate improved from 34.5% to 47.9% between 1990 and 2011 [4].

APL accounts for approximately 5–10% of pediatric AML cases [6], and the vast majority (95–98%) of APL cases are characterized by the presence of the balanced reciprocal translocation t(15;17)(q24;q21), which results in the fusion of the promyelocytic leukemia (*PML*) gene on chromosome 15 with the retinoic acid receptor alpha (*RARA*) gene on chromosome 17. This genetic alteration gives rise to the *PML-RARA* fusion gene, which encodes a chimeric oncoprotein that plays a central role in APL leukemogenesis.

Functionally, the PML-RARA fusion protein disrupts normal myeloid differentiation by acting as an aberrant transcriptional repressor. It binds to retinoic acid response elements (RAREs) on DNA, but, unlike wild-type RARA, it recruits corepressor complexes—such as nuclear receptor corepressor (NCoR), silencing mediator for retinoid and thyroid hormone receptors (SMRT), and histone deacetylases (HDACs)—even in the presence of physiological levels of retinoic acid. This leads to sustained repression of RARA target genes essential for myeloid maturation. Additionally, the fusion protein interferes with the formation and function of PML nuclear bodies (PML-NBs)—subnuclear structures involved in processes such as apoptosis, senescence, and DNA damage response. Disruption of PML-NBs further contributes to leukemogenesis by impairing key tumor suppressor pathways, including p53 activation.

Furthermore, there are rare variant subtypes of APL that do not involve the classic PML-RARA fusion gene generated by the t(15;17)(q24;q21) translocation. Instead, these atypical cases are defined by the presence of alternative RARA fusions with PLZF, NPM1, NuMA, STAT5B or FIP1L1, among others [7,8,9,10,11]. These variant fusions can significantly influence disease phenotype, response to therapy, and prognosis. For instance, PLZF-RARA, resulting from the t(11;17)(q23;q21) translocation, is associated with resistance to all-trans retinoic acid (ATRA) due to the fusion protein’s constitutive repression of retinoic acid target genes and impaired transcriptional activation [8,9]. Similarly, STAT5B-RARA variants exhibit strong transforming ability and are also unresponsive to ATRA or arsenic trioxide (ATO) treatment [10]. Morphologically, variant APL may present with microgranular promyelocytes, where immunophenotype and genetic analyses are crucial for accurate diagnosis. The clinical outcomes for patients with variant APL are generally poorer due to reduced sensitivity to differentiation therapy and lack of standardized treatment protocols [11]. Continued molecular characterization and tailored therapeutic strategies are essential to improve prognosis in these rare and challenging subtypes.

Collectively, these molecular alterations result in a differentiation block at the promyelocyte stage and confer aberrant self-renewal capabilities to the leukemic cells. The consequent accumulation of undifferentiated promyelocytes in the bone marrow and peripheral blood is a hallmark of APL, often accompanied by a life-threatening coagulopathy due to the procoagulant activity of the abnormal cells [12,13,14].

Importantly, the clinical APL experience also underscores the broader interaction between transcription factors, differentiation therapies, and micronutrient homeostasis. The PML-RARA fusion protein contains two zinc finger domains, suggesting a potential role for zinc in modulating transcription factor function [15]. This has led to emerging hypotheses regarding zinc’s involvement in the regulation of leukemic cell differentiation.

Zinc is a fundamental structural element for the proper function of C2H2-type zinc finger proteins (ZFPs), which depend on zinc ions (Zn^2+^) to stabilize their characteristic ββα motifs that are required for DNA binding and transcriptional regulation. In the absence of zinc, key transcription factors such as KLF4, Sp1, and RUNX2 exhibit structural instability and diminished DNA-binding capacity, resulting in impaired transcriptional activity [16]. Zinc has already been shown to play a role in more than 300 enzymatic reactions, thereby serving as a critical regulator of numerous biological processes like metabolic functions, gene expressions, apoptosis and immune modulation. Two major protein families that are involved in zinc homeostasis are the SLC39 family (Zrt/Irt-like proteins, ZIP), facilitating the influx of ions into the cytoplasm, and the SLC30 family (Zinc transporters, ZnT), supporting the efflux of ions from the cytoplasm into other cellular compartments or into the extracellular space [17]. In the case of hematological malignancies, like APL, it was revealed that zinc homeostasis may be disrupted, and altered expression of zinc transporters (e.g., ZIP2, ZIP10, ZnT3) leads to elevated intracellular zinc levels and excessive zinc accumulation [18].

Preliminary preclinical evidence indicates that zinc supplementation may attenuate ATO toxicity by limiting arsenic accumulation in tissues and reducing oxidative damage [19]. Although such strategies are not yet integrated into routine clinical practice and are not part of standard care, they may offer promising directions for adjunctive therapy and toxicity mitigation.

The successful development of targeted therapy for pediatric ALL began with a significant effort to deepen our understanding of its molecular mechanisms [20]. Likewise, unraveling how zinc interacts with leukemic cells, transcription factors, and modulate differentiation therapy in APL may yield adjunctive approaches to either enhance the efficacy or mitigate the toxicity of targeted therapies. In this review, we aim to explore the mechanistic basis of these interrelationships and their potential clinical applications.

## 2. Management of Pediatric APL

The therapeutic approach to APL in children significantly diverges from that of other AML subtypes. APL was the first form of acute leukemia to be effectively treated by the molecular targeting of a transcription factor, with the aim of inducing the differentiation of promyelocytic blasts, rather than relying solely on conventional cytotoxic chemotherapy. A pivotal study published in 1988 by Huang M et al. demonstrated that ATRA could induce the terminal differentiation of APL blasts and achieve complete remission, even in patients who were resistant to conventional chemotherapy [21]. This pioneering clinical experience with the use of ATRA demonstrated the drug’s efficacy and preceded the cloning of RARA, at a time when knowledge of the specific underlying molecular alterations was not yet available to guide its clinical introduction.

Later, it was revealed that ATRA functions by binding to the RARA domain of the PML-RARA fusion oncoprotein, alleviating its repressive effect on gene transcription and thereby facilitating myeloid differentiation [12]. This represented a paradigm shift in oncology, establishing a therapeutic model focused on resolving differentiation arrest rather than directly killing leukemic cells. Nevertheless, the precise molecular basis of this differentiation-inducing strategy—particularly with regard to transcription factor regulation— still remains incompletely elucidated.

Despite its effectiveness in initiating differentiation, ATRA monotherapy is insufficient for the complete eradication of APL, as the disease also involves aberrant self-renewal phenotype in addition to the differentiation block. The PML-RARA fusion protein contributes to leukemogenesis by disrupting the architecture and function of PML-NBs, structures that are crucial for cellular responses to stress and for promoting apoptosis. Consequently, treatment strategies evolved to include chemotherapy in combination with ATRA, which became the standard of care for many years, despite the risk of cytotoxic side effects [13].

More recently, a shift toward chemotherapy-free regimens has been made possible through the introduction of ATO in combination with ATRA as the frontline therapy. ATO exerts its therapeutic effect through multiple mechanisms: it binds directly to the PML-RARA fusion protein, triggering its oxidation and sumoylation, and promoting its degradation via the proteasome. Additionally, ATO acts on wild-type PML, facilitating the reassembly of PML-NBs and enhancing their pro-apoptotic activity. It also indirectly contributes to PML-RARA degradation through activation of caspases and the generation of reactive oxygen species (ROS) [13,22].

While this ATRA–ATO combination has been well validated in adult APL—demonstrating high remission rates, reduced relapse frequency, and comparable survival outcomes to ATRA plus chemotherapy regimens [23]—its use in pediatric populations has only recently begun to be explored. Several clinical trials conducted over the past few years suggested that this chemotherapy-free approach was both safe and effective for children with standard-risk (SR) APL, defined as having a white blood cell (WBC) count below 10,000/μL. In contrast, for high-risk (HR) patients (WBC ≥ 10,000/μL), the inclusion of reduced-dose chemotherapy during the induction phase, alongside ATRA and ATO, appears to be beneficial [24,25,26,27,28]. Taken together, these findings support the adoption of ATRA and ATO as a frontline strategy in pediatric APL, particularly to minimize exposure to anthracyclines, thereby reducing the risk of long-term complications, such as cardiotoxicity.

## 3. Challenges in Differentiation Therapy

Although differentiation therapy has transformed the prognosis of APL—elevating survival rates from previously high mortality to over 90% long-term survival [12,13,14]—the use of ATRA and ATO is not without clinical complications. Both agents are associated with substantial toxicities that often require careful dose adjustments. Two of the most serious adverse events are differentiation syndrome (DS) and fatal hemorrhage, which together contribute significantly to early death in pediatric APL patients. DS typically emerges in 5–20% of pediatric cases, especially following combined ATRA and ATO administration, and carries a mortality rate of up to 5.7% in affected individuals. Concurrently, the incidence of early hemorrhagic death remains between 5–10% [29,30]. ATO, in particular, is known to cause additional side effects such as QT interval prolongation, which poses further clinical risk [13,22].

Moreover, differentiation therapy is rendered less effective in a subset of rare APL-like AML that do not harbor the characteristic PML-RARA fusion gene resulting from the t(15;17) translocation. First identified in the early 1990s, these atypical leukemias—now numbering more than 40 known variants—exhibit morphological and immunophenotypic features similar to APL, but differ substantially in their biological behavior and treatment responsiveness. Notably, they tend to show limited sensitivity to both ATRA and standard chemotherapy regimens [14]. As such, optimizing treatment for APL requires not only pharmacologic strategies, but also supportive interventions and individualized dose modulation to ensure the safety and efficacy of differentiation-based therapies.

## 4. Disrupted Zinc Homeostasis in APL

Acute leukemias are frequently associated with disturbances in zinc metabolism, although the pattern of dysregulation differs between systemic (serum) and intracellular compartments. Clinically, patients with AML often exhibit significant serum zinc deficiency, with median zinc concentrations markedly lower than those observed in healthy individuals (0.68 mg/L vs. 0.86 mg/L, respectively) [31]. In a cohort analysis, more than 75% of AML patients demonstrated subnormal serum zinc levels, frequently accompanied by elevated serum copper concentrations, resulting in an increased copper-to-zinc (Cu/Zn) ratio. This elevated Cu/Zn ratio has been correlated with adverse clinical outcomes and may serve as a prognostic biomarker in AML [31].

Conversely, emerging evidence indicates that leukemic cells may actively accumulate zinc intracellularly. In particular, APL-derived NB4 cells have been shown to possess higher intracellular zinc content and increased expression of specific zinc transporter proteins, such as members of the ZIP and ZnT families (ZIP2, ZIP10, ZnT3), compared to normal peripheral blood cells [18]. The elevated intracellular zinc appears to stabilize the PML-RARA oncoprotein, preventing its degradation; in contrast, zinc depletion in NB4 cells triggers PML-RARA degradation via enhanced caspase-3 activity and increased autophagy, indicating its role in maintaining leukemic cell survival.

Paralleling these mechanistic insights, comprehensive gene expression analyses in AML—including NB4 and U937 cell lines—have revealed subtype-specific dysregulation of zinc homeostasis genes: increased expression of ZIP4 and reduced expression of ZnT5 and ZnT7 are associated with inferior survival outcomes in AML patients [30]. Functional studies further showed that zinc chelation by N,N,N′,N′-tetrakis(2-pyridylmethyl)ethylenediamine (TPEN) in NB4 cells induces apoptosis while altering transcription of multiple ZIP and ZnT genes [32]. Overall, these findings suggest that intracellular zinc accumulation is not merely a bystander phenomenon, but rather contributes to APL pathogenesis through maintenance of the oncogenic fusion protein and regulation of differentiation and apoptotic pathways.

This paradoxical pattern and dual dysregulation of zinc—i.e., deficiency in the extracellular milieu and excess within leukemic blasts—suggests a reprogramming of zinc homeostasis in leukemic cells and is gaining recognition as a potential therapeutic target. By modulating zinc levels or transporter activity, it may be possible to sensitize leukemic cells to treatment, alleviate chemotherapy-related toxicity, or counteract drug resistance mechanisms [33]. These findings open new strategies for the development of adjunctive therapies in APL management, particularly in the context of precision medicine and metal ion-targeted therapies.

## 5. Role of Zinc in Oncoprotein Stability in APL

In the context of APL, zinc has been shown to be essential for maintaining the structural and functional stability of oncoproteins, especially the PML–RARA fusion protein, a key oncoprotein driving leukemogenesis [34]. Experimental models using the NB4 APL cell line revealed that chelation of intracellular zinc using TPEN leads to time-dependent degradation of PML-RARA. This degradation occurs via activation of apoptotic pathways, underscoring the zinc-dependent stability of this fusion protein [35]. Under conditions of zinc depletion, leukemic cells exhibit increased phosphorylation of stress-related kinases, including p38 MAPK and JNK, while concurrently showing downregulation of survival pathways such as Akt/mTOR. Notably, these stress-induced effects can be reversed by supplementation with exogenous zinc or nitric oxide (NO). Furthermore, clinical data support a role for metallothionein 2A (MT2A)—a zinc-binding protein—in modulating these pathways; elevated MT2A expression correlates positively with survival signaling and inversely with stress pathway activation [36].

Consistent with these findings, a more recent study demonstrated that sustained zinc deficiency in NB4 cells results in increased caspase-3 activity, which facilitates the degradation of PML–RARA. Restoration of zinc levels reversed this process, reaffirming the oncoprotein’s zinc dependency. Interestingly, analogous effects have been observed in chronic myeloid leukemia (CML) models: in K562 cells, zinc deprivation similarly promoted degradation of the BCR–ABL1 fusion protein [18]. These observations suggest a broader requirement for intracellular zinc in stabilizing oncogenic fusion proteins across different leukemia subtypes.

In addition to its impact on oncoproteins, zinc imbalance influences the expression of zinc transporters. For instance, in response to zinc depletion, AML cells—including APL-derived NB4 cells—upregulate transporters such as ZIP2, ZIP10, and ZnT3, likely as a compensatory mechanism to restore intracellular zinc homeostasis [18].

Overall, the intracellular zinc equilibrium appears to be a critical determinant of leukemia cell fate, influencing survival, apoptosis, and protein stability. These findings propose that therapeutic manipulation of zinc levels could represent a novel strategy to enhance treatment efficacy in APL by targeting oncoprotein stability and stress-response signaling.

## 6. Dual Effects of Zinc: Therapeutic Implications

**Figure 1:**
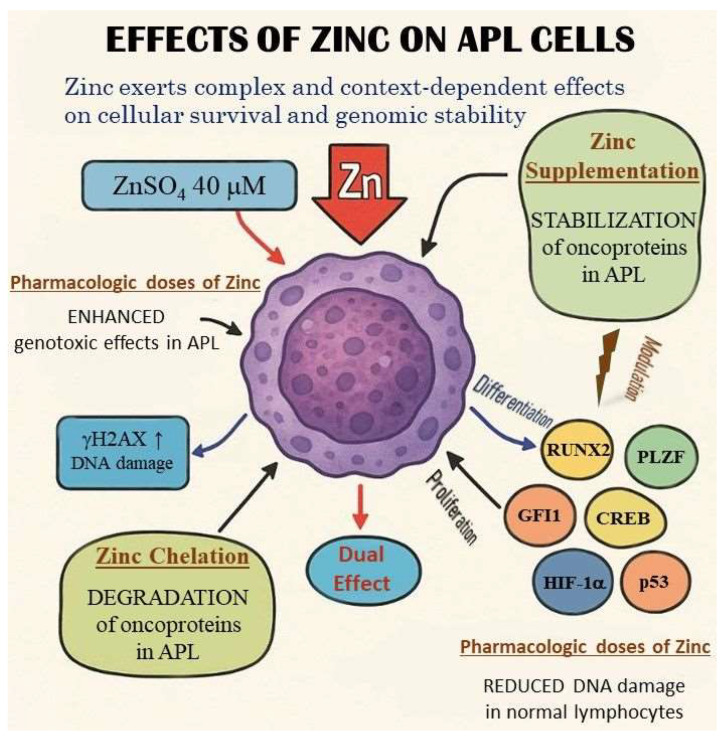
Dual effects of zinc on APL cells. Dual and context-dependent effects of zinc—protective by reducing DNA damage in normal cells and cytotoxic by enhancing genotoxic effects in leukemic blasts—are schematically depicted. The differences between normal and malignant cell phenotypes, along with the opposing modulatory effects of the highlighted factors, warrant particular attention. The distinct mechanisms of zinc chelation and supplementation are also illustrated, highlighting the dual role of zinc in modulating cellular processes.

Zinc exerts complex and context-dependent effects on cellular survival and genomic stability, particularly within leukemic versus normal hematopoietic cells. Notably, its influence can diverge dramatically between malignant and non-malignant cells. Experimental evidence demonstrates that supplementation with moderate concentrations of zinc sulfate (ZnSO_4_, 40 μM) exacerbates genotoxic and cytotoxic effects in AML cells, particularly under oxidative stress conditions induced by hydrogen peroxide (H_2_O_2_) or ultraviolet (UV) exposure. In these AML cells, zinc increases γH2AX expression, a marker of persistent DNA double-strand breaks and damage signaling. By contrast, the same treatment in normal lymphocytes leads to reduced DNA damage and enhanced repair efficiency, indicating a protective effect in non-leukemic contexts (Figure 1) [37].

Dual and context-dependent effects of zinc—protective by reducing DNA damage in normal cells and cytotoxic by enhancing genotoxic effects in leukemic blasts—are schematically depicted. The differences between normal and malignant cell phenotypes, along with the opposing modulatory effects of the highlighted factors, warrant particular attention. The distinct mechanisms of zinc chelation and supplementation are also illustrated, highlighting the dual role of zinc in modulating cellular processes.

Moreover, zinc deficiency in normal cells has been shown to impair DNA repair processes and promote the accumulation of DNA damage, although overall gene expression profiles remain largely unchanged across varying zinc levels in non-malignant cells [37]. These findings highlight a potential therapeutic window, wherein modulating zinc levels could selectively exploit vulnerabilities in leukemic cells without harming normal counterparts.

Given prior observations that intracellular zinc depletion leads to oncoprotein degradation (e.g., PML–RARA in APL) and apoptosis in leukemic cells, the dual behavior of zinc opens two promising investigational avenues. First, zinc chelation strategies may be used to destabilize zinc-dependent transcription factors and fusion oncoproteins, selectively triggering apoptosis in leukemia cells. Second, pharmacologic zinc supplementation, when combined with DNA-damaging agents, could increase leukemic cell sensitivity by enhancing genotoxic stress, while concurrently supporting genome integrity in normal hematopoietic cells (Figure 1).

These opposing yet therapeutically exploitable effects underscore the need for further mechanistic and translational studies to define optimal zinc modulation strategies in leukemia. Such investigations may establish zinc as both a biological marker and a dual-function therapeutic tool in the precision treatment of hematologic malignancies.

## 7. Regulation of Transcription Factors by Zinc in APL Development and Therapy

### 7.1. Zinc-Dependent Transcription Factors

#### 7.1.1. Runt-Related Transcription Factor 2 (RUNX2)

RUNX2, a zinc-finger (ZnF) transcription factor, is known for promoting osteoblast differentiation in mesenchymal stem cells (MSCs) via signaling pathways such as BMP-2/Smad-1 and cAMP–PKA–CREB, both of which are indirectly regulated by zinc via signaling modulation. Experimental results showed that zinc deficiency in osteoblasts markedly reduced the expression of BMP-2 and Smad-1 which consequently led to a decrease in that of RUNX2 [38]. Likewise, zinc was proved to activate the cAMP–PKA–CREB pathway through upstream signaling mechanisms via G-protein coupled receptor GPR39, which binds extracellular zinc and triggers G-protein signaling, causing a rise in intracellular cAMP and subsequent PKA activation and CREB phosphorylation [39,40,41]. Although primarily an osteogenic regulator, RUNX2 is also implicated in leukemogenesis: its mRNA—alongside RUNX1/3—is elevated in AML cell lines, and mouse models showed that RUNX2 cooperates with the CBFβ-SMMHC fusion protein in leukemic transformation [42,43]. Cases of AML with RUNX2 haploinsufficiency further support its oncogenic role in AML development [44]. In APL contexts, ATRA treatment modestly increases RUNX2 expression in NB4 cells, while non-APL AML lines like HL-60 show stronger upregulation under ATRA, indicating lineage promiscuity [45]. However, in APL, RUNX2 is transcriptionally silenced during early leukemogenesis via PML-RARA–driven chromatin reconfiguration—shifting RUNX2 from active (A) to repressive (B) compartments [46]. Further, genome-wide profiling in NB4 cells revealed RUNX2 among genes associated with apoptosis regulation following ATRA or ATO exposure [47]. The Promyelocytic Leukemia Zinc Finger (PLZF) transcription factor also acts upstream of RUNX2 and is itself zinc-regulated [48], by which zinc levels could influence the RUNX2 axis in APL. Altering RUNX2 activity by modulating zinc availability may therefore affect proliferation and differentiation of APL cells.

#### 7.1.2. Krupple-like Factor 4 (KLF4)

KLF4 is a C2H2 ZnF transcription factor with dual roles in APL proliferation. On the one hand, functional studies using CRISPR/Cas9 in NB4 and MonoMac6 cells showed that deletion of KLF4 reduces proliferation and increases apoptosis, especially under ATRA-induced differentiation conditions, indicating that endogenous KLF4 supports leukemic survival and resistance to differentiation therapy [49]. High KLF4 levels were also proven to be associated with poor prognosis in pediatric Burkitt lymphoma through repression of DYRK2 tyrosine kinase which is known for promoting proteosomal degradation and depletion of c-Myc and activation of p53 [49].

While, on the other hand, low expression levels of KLF4 were also found in B-cell non-Hodgkin and Hodgkin lymphomas, multiple myeloma, and CDX2-driven AML, which—considering its otherwise normal expression level—suggests its potential tumor-suppressive function [50]. In the latter case, it has been demonstrated that the use of PPARγ agonists reversed the deregulation of this signaling pathway and led to leukemic cell death in CDX2 AML cells [51].

To further substantiate its tumor suppressor role, it has also been revealed that KLF4 expression is silenced in pediatric T-ALL by CpG promoter methylation with aberrant expression of MAP2K7 [52,53], and that KLF4 was identified as an early down-regulated gene in PML-RARA-mediated leukemogenesis [46]. Moreover, KLF4 was also shown to promote cell senescence through a complex network of miR-203, survivin and p21 [54] and its interplay with the JAK/STAT signaling pathway further illustrated its context-dependent functions in leukemia [55,56].

In addition to the above, it has also been demonstrated that ATRA exerts an inducing effect on KLF4 expression levels via RARA recruitment to GC-rich promoters in cooperation with transcription factor Sp1, which is a ubiquitously expressed ZnF factor playing an important role in cell growth, differentiation, and apoptosis [57]. In addition, the application of the small-molecule ML-133 metal chelator further contributed to this effect by modulating intracellular labile zinc homeostasis, thereby inducing the expression of KLF4 [58].

Thus, zinc plays a context-dependent role in regulating the survival of leukemic cells, determined by the expression level of KLF4, as well as by the cell type–specific pattern of its interaction partners.

#### 7.1.3. Promyelocytic Leukemia Zinc Finger (PLZF/ZBTB16/ZFP145)

PLZF is a ZnF transcription factor originally identified due to its involvement in the rare PLZF-RARA fusion-positive APL variant resulting from t(11;17), which exhibits ATRA resistance [59]. In normal MSCs, PLZF positively regulates osteogenic differentiation via activation of RUNX2 and downstream osteogenic gene expression; loss of PLZF impairs bone formation, while overexpression stimulates RUNX2 and osteogenic markers [48,60]. Since PLZF and RUNX2 are both zinc-dependent, zinc could support a PLZF→RUNX2 axis that promotes differentiation. In APL, modulating zinc to enhance PLZF expression or activity may improve ATRA responsiveness by supporting differentiation pathways. Another potential modulatory mechanism by which zinc may overcome ATRA resistance is its known effect of activating PKA [40], since it has been shown in mouse models that combining ATRA with a cAMP analogue promotes PKA activation, which in turn enhances PLZF/RARA degradation and facilitates differentiation [61].

#### 7.1.4. Zinc Finger Protein 521 (ZNF521/ZFP521)

ZNF521 is a multi–ZnF transcription factor essential for hematopoietic stem cell self-renewal and differentiation blockade. Its overexpression is associated with enhanced proliferation in AML subtypes, notably MLL-AF9-positive AML, where it cooperates with MLL fusion proteins [62]. On the one hand, enforced expression of ZNF521 preserves the self-renewing and primitive state of progenitors. On the other hand, loss of ZNF521 accelerates differentiation into myeloid and lymphoid lineages. These finding makes ZNF521 a candidate onco-driver in leukemias that involve differentiation block. Importantly, ATRA downregulates ZNF521 expression in monocytic AML models (e.g., THP-1, NOMO-1), promoting differentiation [63]. This suggests that zinc modulation—by regulating ZNF521 stability or expression—may amplify ATRA’s differentiation-inducing effect and suppress proliferation of APL or AML blasts. ATRA-induced downregulation is supposed to be indirect, occurring via differentiation-linked pathways or epigenetic reprogramming, as RAR/RXR binding sites (RARE) have not been reported in promoter studies [63,64].

#### 7.1.5. Specificity Protein 1 (Sp1)

Sp1 is a ubiquitous C2H2 ZnF transcription factor crucial in regulating genes related to cell cycle, survival, and differentiation. In APL NB4 cells, low-dose arsenic exposure produces ROS-induced oxidation of Sp1, altering expression of a subset of oncogenic targets [57,65,66]. Although this mechanism accounts for limited gene expression changes (~26%), it illustrates Sp1’s susceptibility to redox modification. In CD34^+^ AML patients, Sp1 and c-Myc are co-upregulated with survivin, contributing to leukemic stem cell resistance [67]. Since Sp1 activity depends on zinc and modulates survivin and other survival genes [66], combining zinc modulation with Sp1 inhibition could impair leukemic proliferation and enhance therapy response. The redox sensitivity of Sp1 warrants greater emphasis regarding its therapeutic implications, with suggestions for combinatorial strategies such as zinc chelation combined with ATO to enhance oxidative Sp1 inactivation.

#### 7.1.6. Growth Factor Independence 1 (GFI1)

**Figure 2:**
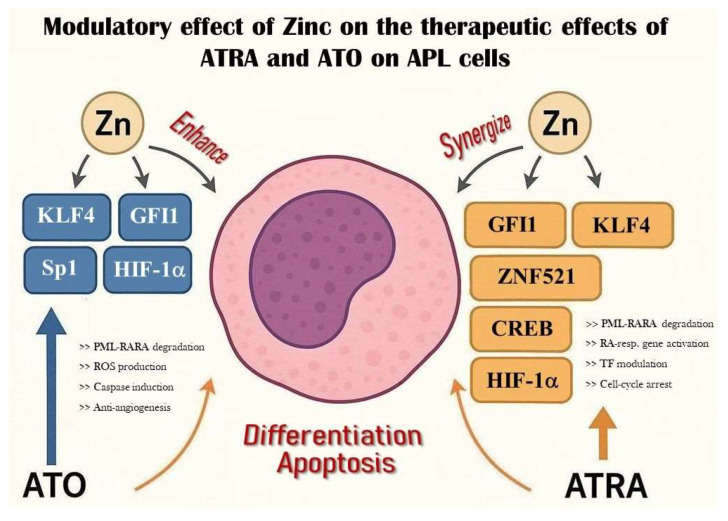
Modulatory effect of zinc on ATO and ATRA therapy in APL. The figure depicts the mechanisms by which ATO and ATRA act on APL cells to promote differentiation or apoptosis via key transcription factors (KLF4, GFI1, HIF1α, Sp1, CREB, ZNF521). Zinc exerts context-dependent modulatory effects based on tissue distribution and expression. Potential therapeutic strategies arising from these interactions are detailed in Table 1.

GFI1 is a nuclear transcriptional repressor containing six C2H2 zinc fingers and is directly activated by PML-RARA at a super-enhancer region in APL cells [47,68]. It was first discovered in the hematopoietic system as a key regulator of stem cell homeostasis and neutrophil/T-cell development; however, it is also essential for inner-ear hair and intestinal secretory cell or sensory neuron differentiation [69]. GFI1 expression is essential for maintaining the undifferentiated, proliferative phenotype in APL; its knockdown induces partial myeloid differentiation, cell cycle arrest, apoptosis, and suppression of leukemic propagation in vivo [47]. By degrading PML-RARA with ATRA/ATO, GFI1 levels likely decrease, releasing repression and promoting differentiation; however, GFI1 defects, especially low gene expressions, were also reported to promote the development of malignancy, which raises the need for further investigations. Specific mutations or germline GFI1 variants alter DNA-binding specificity or transcriptional repression and therefore may lead to different effects and predispose to certain diseases (severe congenital neutropenia, AML). Accordingly, the GFI1 36S variant is inhibited by the AML1/ETO fusion in t(8;21) AML, reducing its repressive function. However, the rare GFI1 36N variant does not interact with AML1/ETO, so its repressor function remains active [70,71]. Given its reliance on ZnF domains, zinc availability may support GFI1’s function in maintaining APL proliferation—implying that zinc deprivation could reduce GFI1 activity and impair leukemic cell growth (see Figure 2 and Table 1).

### 7.2. Zinc-Modulated Non–Zinc Finger Factors

#### 7.2.1. cAMP Response Element Binding Protein (CREB)

CREB is a basic leucine zipper transcription factor frequently overexpressed in AML, including APL, and correlates with poor prognosis [72]. In MSCs, zinc activates CREB via the cAMP–PKA pathway, which subsequently induces RUNX2 expression and osteogenic differentiation [40]. In APL, similar activation of CREB by zinc may enhance ATRA-induced differentiation through RUNX2 upregulation. However, CREB also promotes survival in leukemic cells by upregulating anti-apoptotic genes (e.g., *BCL-2*, *MCL-1*) and proliferation drivers (e.g., cyclins A1/D2), and protects APL cells against apoptosis—even under ATRA-resistant conditions [73,74]. In experimental models, it has also been revealed that CREB overexpression stimulates C/EBPδ (a differentiation regulator), which blocks normal granulocytic differentiation and contributes to the development of myeloid leukemogenesis, by which its silencing may trigger apoptosis and slows proliferation [74]. Thus, zinc-induced CREB activity may have a dual effect: fostering differentiation via RUNX2 activation, but simultaneously supporting leukemic cell survival. Thus, in this context, experimental approaches such as zinc dose titration could be proposed to dissect its opposing roles in differentiation and survival, and zinc dosing in APL therapy must therefore be carefully calibrated.

#### 7.2.2. Hypoxia-Inducible Factor 1-Alpha (HIF-1α)

HIF-1α is a non-ZnF transcription factor that becomes stabilized and activated under hypoxic conditions, such as those present in the bone marrow niche. It plays a critical role in sustaining the leukemic phenotype in APL by promoting survival, stemness, and therapy resistance. Elevated expression of HIF-1α in AML, including APL, is associated with poor prognosis and supports leukemic stem cell maintenance within hypoxic microenvironments [75]. HIF-1α contributes to the survival of APL cells by inhibiting apoptosis, including resistance to ATO-induced cell death. Importantly, HIF-1α also regulates the expression of survival-promoting genes and supports metabolic adaptations that help leukemic cells persist under stress. Despite these oncogenic effects, HIF-1α can paradoxically cooperate with differentiation-promoting transcription factors such as C/EBPα, RUNX1, and PU.1 to induce myeloid maturation under specific conditions, and its inhibition of oncogenic regulators like c-MYC, miR-17, and miR-20a further supports differentiation [75].

Although HIF-1α itself is not a zinc finger protein, its activity may still be indirectly influenced by zinc availability. Zinc is known to affect oxidative stress and cellular redox states, both of which modulate HIF-1α stability and transcriptional activity. Therefore, zinc deficiency may increase ROS, stabilizing HIF-1α even under normoxic conditions, thus potentially exacerbating its pro-leukemic effects. On the other hand, zinc supplementation in human solid tumor cells such as prostate and glioblastoma has been shown to downregulate HIF-1α levels and activity [76]. It was demonstrated that adding zinc to hypoxic cancer cells induced proteasomal degradation of HIF-1α. In contrast, adequate zinc levels might help regulate ROS and reduce inappropriate HIF-1α activation. Since direct evidence in APL is limited, in the future, further validation experiments are suggested to delineate the potential therapeutic effect of zinc to influence HIF-1α pathways through redox balance, which may serve as a novel approach for modulating APL proliferation and therapy resistance [75].

#### 7.2.3. p53

p53, a well-known and extensively investigated tumor suppressor transcription factor requiring zinc for proper folding and DNA binding, regulates cell cycle arrest, apoptosis, and DNA repair. Mutations impairing zinc coordination (e.g., R175H) compromise its function, which is common in many cancers [77,78]. In APL, Metallothionein 1G (MT1G)—a zinc-binding protein—has been shown to inhibit p53-dependent differentiation by sequestering intracellular zinc in NB4 cells, thereby impairing ATRA-induced cytodifferentiation [79]. Overexpression of MT1G correlates with poor differentiation outcomes. Thus, intracellular zinc levels directly modulate p53 activity and, consequently, the proliferation or differentiation status of APL cells. This further underscores the clinical potential and significance of targeting MT1G-mediated zinc sequestration to restore p53 function, which may link this approach to existing p53-reactivating therapies. All of these points, altogether, strongly emphasize that maintaining zinc availability may support p53 function and enhance ATRA efficacy (see Figure 2 and Table 1).

## 8. Discussion and Future Directions

Although implementation of differentiation-induction strategy with ATRA+/-ATO resulted in a vast improvement of treatment outcome measures in APL, the still substantial rates of treatment-related morbidity and mortality, and treatment resistance warrant further approaches to enhance survival rates in this patient group. Increasing efficacy and safety of ATRA+/-ATO differentiation induction regimens by additional biologic response modifiers may contribute to the desired goal.

The present review offers comprehensive molecular insights into the crucial role of zinc in regulating the stability of fusion proteins, transcription factor activity, and leukemic cell fate. Here, we highlight zinc homeostasis, from influencing the stability of oncoproteins to modulating essential transcriptional regulators. Zinc exerts complex and context-dependent effects on cellular survival and genomic stability, particularly within leukemic versus normal hematopoietic cells. It has been revealed that intracellular zinc accumulation supports oncogenic signaling by stabilizing zinc finger transcription factors (e.g., RUNX2, KLF4, Sp1, GFI1) and fusion proteins, while zinc depletion triggers apoptosis and oncoprotein degradation. In contrast, extracellular zinc levels are typically deficient in AML patients and may impact normal hematopoietic function. These dual and context-dependent effects of zinc—protective in normal cells and cytotoxic in leukemic blasts—offer a unique and promising therapeutic window. The body of current literature may support the notion that the regulation of zinc can be exploited in two beneficial ways for the benefit of patients. First, there are pieces of evidence suggesting that zinc may sensitize APL (or variant APL) cells to regulatory actions exerted by other factors, i.e., ATRA/ATO. Secondly, regulation exerted by zinc may improve the function of healthy cells required for important body defense mechanisms (i.e., optimal immune response, intact mucous membranes).

These findings, along with clinical observations and molecular interactions, highlight multiple therapeutic opportunities for targeting zinc homeostasis. First, zinc chelation using agents such as TPEN may destabilize zinc-dependent fusion oncoproteins, while zinc supplementation could be employed to selectively enhance genotoxic stress in malignant cells. Furthermore, targeting aberrant expression of zinc transporters such as ZIP2, ZIP10, and ZnT3 and their pathways offers a promising strategy to influence intracellular zinc levels and sensitize leukemic cells to treatments. In addition, zinc also plays a crucial role in the structural integrity and activity of multiple transcription factors—including RUNX2, KLF4, ZNF521, Sp1, and GFI1—that are essential for leukemogenesis and differentiation block. Modulating zinc availability may therefore allow indirect control over these transcriptional regulators and shift the balance from leukemic proliferation toward terminal differentiation. Such modulation may also enhance the efficacy of ATRA and ATO by restoring transcriptional responses in differentiation processes. Finally, combination therapies that integrate zinc modulation with differentiation agents and redox-targeted drugs represent another compelling direction. This strategy could address both the resistance seen in variant or high-risk APL subtypes and the toxic side effects associated with conventional differentiation induction and combined chemotherapy. Simultaneously, serum zinc levels and the copper/zinc ratio could serve as accessible, cost-effective biomarkers for risk stratification, treatment response monitoring, or early relapse prediction.

In conclusion, zinc’s dual role in leukemic cell biology—as both a structural cofactor for oncogenic proteins and a modulator of redox balance—positions it as a promising adjunctive target in the treatment of APL. Future investigations should define the optimal dose, timing, and context for such zinc-focused interventions, carefully balancing zinc’s dual effects to avoid inadvertently supporting leukemic survival while maximizing anti-leukemic efficacy. As clinical data in this area remain limited, based on the molecular background and experimental findings, in the future, well-designed clinical trials incorporating zinc-chelating therapies may potentially contribute to overcome ATRA resistance and achieve more effective treatment of the disease.

## Figures and Tables

**Table 1:** Regulation of Transcription Factors by Zinc in APL Development and Therapy.

	Molecular Targets	Function and Role	Effect on APL Proliferation/Differentiation	Effect of Zinc on TF and APL Proliferation	Therapeutic Strategy	Reference
ZnF Transcription Factors	RUNX2	ZnF transcription factor regulating osteogenesis and cell fate via cAMP–PKA–CREB and BMP–Smad pathways	Contributes to leukemogenesis; upregulated in AML; silenced in APL via chromatin repression; modestly induced by ATRA in NB4 cells.	Zinc activates RUNX2 via BMP-2 and CREB pathways; may support differentiation in APL under ATRA/ATO treatment	Zinc chelation to destabilize RUNX2	[38,39,40,41,42,43,44,45,46,47]
	KLF4	C2H2-type ZnF transcription factor involved in cell cycle regulation and differentiation	Dual role: supports leukemic survival (steady state) but acts as tumor suppressor when overexpressed; represses ATRA-induced apoptosis unless silenced	Zinc stabilizes KLF4 and enhances its DNA binding; may enhance ATRA effect via co-activation with Sp1.	Zinc titration to find the dose synergizing with ATRA	[46,49,57]
	PLZF (ZBTB16)	ZnF transcription factor; component of PLZF–RARA fusion in APL variant	PLZF–RARA fusion leads to ATRA resistance; blocks differentiation; wild-type PLZF supports osteogenic differentiation.	Zinc modulates PLZF activity; loss impairs differentiation; zinc may enhance MSC differentiation via PLZF→RUNX2 axis.	Zinc chelation to modulate PLZF and destabilize RUNX2	[48,59,60]
	ZNF521	ZnF transcription factor maintaining stemness of HSCs; blocks differentiation.	Highly expressed in early progenitors and MLL-rearranged AML; inhibits differentiation; downregulated by ATRA.	Zinc stabilizes ZNF521 structure; role not directly defined, but zinc may modulate differentiation response through ZNF521 repression.	Zinc supply combined with ATRA to induce downregulation of ZNF521	[62,63]
	Sp1	Ubiquitous C2H2 ZnF transcription factor regulating survival, cell cycle, and differentiation genes.	Promotes survival of AML cells; oxidation by ATO suppresses oncogenic gene expression; interacts with KLF4 and survivin pathways.	Zinc essential for Sp1’s structural stability; zinc may synergize with ATRA/ATO or Sp1 inhibition for anti-leukemic effects.	Zinc chelation with ATO to enhance oxidative inactivation of Sp1	[57,65,66,67]
	GFI1	ZnF transcription factor acting as transcriptional repressor.	Upregulated by PML–RARA in APL; maintains leukemic state; knockdown promotes differentiation and apoptosis.	GFI1 function depends on ZnF integrity; zinc levels may affect its DNA binding and repression capacity, though not yet experimentally confirmed.	Zinc supply combined with ATRA/ATO to decrease expression of GFI1	[47,68]
Non-ZnF Transcription Factors	CREB	Non-ZnF transcription factor activated via cAMP–PKA pathway.	Overexpressed in AML; promotes survival and blocks differentiation; supports APL therapy resistance.	Zinc activates cAMP–PKA–CREB signaling; may enhance APL survival or promote differentiation depending on dose and context.	Zinc titration to find the dose promoting differentiation	[40,72,73,74]
	HIF-1α	Non-ZnF transcription factor activated under hypoxia.	Promotes stemness, therapy resistance, and anti-apoptotic pathways in APL; paradoxically also supports myeloid differentiation in some contexts.	Zinc influences ROS and redox balance, which regulate HIF-1α stability; indirect modulation of APL proliferation possible.	Zinc supply to balance cellular redox states and downregulate HIF-1α	[75,76]
	p53	Non-ZnF tumor suppressor TF regulating apoptosis, DNA repair.	Suppresses APL proliferation; required for ATRA-induced differentiation; loss impairs response to therapy.	Zinc critical for structural stability of p53; deficiency causes misfolding and dysfunction; zinc sequestration by MT1G impairs p53 in APL.	Zinc chelation to restore p53 function	[77,78,79]

Summary of the key transcription factors involved, their roles in APL development and progression, and the modulatory effects of zinc, accompanied by relevant supporting literature references.

## Data Availability

We confirm that neither the manuscript nor any parts of its content are currently under consideration for publication with or published in another journal.
